# Social rhythms and occupational functioning disturbance in remitted bipolar patients

**DOI:** 10.1192/j.eurpsy.2021.1654

**Published:** 2021-08-13

**Authors:** A. Ben Cheikh Ahmed, U. Ouali, H. Jemli, A. Aissa, F. Nacef

**Affiliations:** Department Psychiatry A, Razi Hospital, Manouba, Tunisia

**Keywords:** chronobiology, social rhythms, occupational functioning, Bipolar Disorders

## Abstract

**Introduction:**

Biological rhythm disturbance is etiologically involved in mood disorders. Previous literature focused on studying sleep disruption in bipolar disorders (BD). However, only a few studies addressed the influence of social rhythms and occupational functioning as they may affect circadian regularity and consequently be a critical pathway to mood symptoms.

**Objectives:**

The main aim of this study was to assess biological rhythms in remitted bipolar patients and to evaluate their social rhythms and occupational functioning.

**Methods:**

We recruited a total of 80 euthymic outpatients with BD and 80 control subjects. Biological rhythm disruptions were assessed using the Biological Rhythm Interview of Assessment in Neuropsychiatry (BRIAN), an interviewer administered questionnaire that assesses disruptions in sleep, eating patterns, social rhythms, and general activity.

**Results:**

Patients with BD experienced greater biological rhythm alterations than the control group (BRIAN total scores 35.26±9.21 vs. 25.84±2.68). In addition to their sleep-wake rhythm (mean scores 11.1±3.95 vs. 7.41±1.41), patients were particularly more impaired than the control group with regards to social rhythms (7.31 ± 2.57 vs. 5.24 ± 1.06) and general activity (8.9 ± 3.35 vs. 7.01 ± 1.4).

**Conclusions:**

Our study indicated that patients with BD experience major disruptions in their social rhythms and occupational functioning. These alterations may lead to unstable biological rhythms and to a higher risk of mood episodes. Therefore, consolidating social rhythms and functioning appears to be a crucial step for preventing relapses in patients with BD.

**Disclosure:**

No significant relationships.

